# Supplementary material for: Clinical readiness for essential maternal and child health services in Kenya: A cross-sectional survey
Source: PLOS Glob Public Health. 2023 Dec 15;3(12):e0002695. doi: 10.1371/journal.pgph.0002695 (PMC10723700; doi:10.1371/journal.pgph.0002695)
Supplement: S1 Table — (DOCX) [file pgph.0002695.s001.docx]

**S1 Table. Facilities in Homa-Bay, Kisumu and Migori counties included in study.**

| **Facility Name** | **Facility County** | **Facility Level** |
| --- | --- | --- |
| Homa Bay County Teaching and Referral Hospital | Homa-Bay | Level 5 |
| Kabondo Sub County Hospital | Homa-Bay | Level 4 |
| Kandiege Sub County Hospital | Homa-Bay | Level 4 |
| Kendu Sub County Hospital | Homa-Bay | Level 4 |
| Kisegi Sub County Hospital | Homa-Bay | Level 4 |
| Marindi Sub County Hospital | Homa-Bay | Level 4 |
| Mbita Sub County Hospital | Homa-Bay | Level 4 |
| Ndhiwa Sub County Hospital | Homa-Bay | Level 4 |
| Ogongo Sub County Hospital | Homa-Bay | Level 4 |
| Rachuonyo Sub County Hospital | Homa-Bay | Level 4 |
| Rangwe Sub County Hospital | Homa-Bay | Level 4 |
| Suba Sub County Hospital | Homa-Bay | Level 4 |
| Jaramogi Oginga Odinga Teaching and Referral Hospital | Kisumu | Level 5 |
| Kisumu County Hospital | Kisumu | Level 5 |
| Ahero Sub County Hospital | Kisumu | Level 4 |
| Chulaimbo Sub County Hospital | Kisumu | Level 4 |
| Gita Sub County Hospital | Kisumu | Level 4 |
| Kombewa Sub County Hospital | Kisumu | Level 4 |
| Manyuanda Sub County Hospital | Kisumu | Level 4 |
| Masogo Sub County Hospital | Kisumu | Level 4 |
| Migosi Sub County Hospital | Kisumu | Level 4 |
| Miranga Sub County Hospital | Kisumu | Level 4 |
| Muhoroni Sub County Hospital | Kisumu | Level 4 |
| Nyahera Sub County Hospital | Kisumu | Level 4 |
| Nyakach Sub County Hospital | Kisumu | Level 4 |
| Nyang'oma Sub County Hospital | Kisumu | Level 4 |
| Rabuor Sub County Hospital | Kisumu | Level 4 |
| Migori County Hospital | Migori | Level 5 |
| Awendo Sub County Hospital | Migori | Level 4 |
| Isibania Sub County Hospital | Migori | Level 4 |
| Karungu Sub County Hospital | Migori | Level 4 |
| Kegonga Sub County Hospital | Migori | Level 4 |
| Kuria Sub County Hospital | Migori | Level 4 |
| Macalder Sub County Hospital | Migori | Level 4 |
| Ntimaru Sub County Hospital | Migori | Level 4 |
| Othoro Sub County Hospital | Migori | Level 4 |
| Rongo Sub County Hospital | Migori | Level 4 |
