# Supplementary material for: Clinical readiness for essential maternal and child health services in Kenya: A cross-sectional survey
Source: PLOS Glob Public Health. 2023 Dec 15;3(12):e0002695. doi: 10.1371/journal.pgph.0002695 (PMC10723700; doi:10.1371/journal.pgph.0002695)
Supplement: S2 Table — (DOCX) [file pgph.0002695.s002.docx]

**S2 Table.** **Provider Perception of Supply Availability by Obstetric and Neonatal Interventions and Intraclass Correlation Coefficient (ICC).**

| **Supply Availability** | **Never available** | **Rarely available** | **Sometimes available** | **Always available** | **I don't know** | **Intraclass correlation coefficient (ICC)** |
| --- | --- | --- | --- | --- | --- | --- |
| **General supplies** |  |  |  |  |  |  |
| Disposable gloves | 0 (0.0%) | 2 (1.5%) | 12 (9.1%) | 118 (89.4%) | 0 (0.0%) | 0.055 |
| Blood pressure cuff | 1 (0.8%) | 1 (0.8%) | 17 (12.9%) | 112 (84.8%) | 1 (0.8%) | 0.050 |
| Stethoscope | 3 (2.3%) | 7 (5.3%) | 25 (18.9%) | 96 (72.7%) | 1 (0.8%) | 0.063 |
| IV kits | 0 (0.0%) | 1 (0.8%) | 17 (12.9%) | 114 (86.4%) | 0 (0.0%) | 0.000 |
| IV poles | 0 (0.0%) | 3 (2.3%) | 7 (5.3%) | 121 (91.7%) | 1 (0.8%) | 0.025 |
| IV fluids | 0 (0.0%) | 0 (0.0%) | 15 (11.4%) | 117 (88.6%) | 0 (0.0%) | 0.375 |
| Urinary catheters | 0 (0.0%) | 2 (1.5%) | 40 (30.3%) | 90 (68.2%) | 0 (0.0%) | 0.443 |
| Oxygen source | 7 (5.3%) | 6 (4.5%) | 43 (32.6%) | 75 (56.8%) | 1 (0.8%) | 0.244 |
| **Pre-eclampsia/eclampsia supplies** |  |  |  |  |  |  |
| Urine dipstick | 11 (8.3%) | 15 (11.4%) | 53 (40.2%) | 50 (37.9%) | 3 (2.3%) | 0.023 |
| Magnesium sulfate | 0 (0.0%) | 4 (3.0%) | 15 (11.4%) | 113 (85.6%) | 0 (0.0%) | 0.000 |
| Diazepam | 7 (5.3%) | 12 (9.1%) | 55 (41.7%) | 58 (43.9%) | 0 (0.0%) | 0.236 |
| Parenteral anti-hypertensives | 7 (5.3%) | 9 (6.8%) | 44 (33.3%) | 71 (53.8%) | 1 (0.8%) | 0.071 |
| Calcium gluconate | 13 (9.8%) | 11 (8.3%) | 35 (26.5%) | 73 (55.3%) | 0 (0.0%) | 0.233 |
| **Postpartum hemorrhage supplies** |  |  |  |  |  |  |
| Oxytocin | 0 (0.0%) | 0 (0.0%) | 5 (3.8%) | 127 (96.2%) | 0 (0.0%) | 0.000 |
| Ergometrine | 76 (57.6%) | 11 (8.3%) | 22 (16.7%) | 20 (15.2%) | 3 (2.3%) | 0.276 |
| Intrauterine balloon tamponade | 12 (9.1%) | 7 (5.3%) | 19 (14.4%) | 93 (70.5%) | 1 (0.8%) | 0.154 |
| Non-pneumatic anti-shock garment | 103 (78.0%) | 3 (2.3%) | 7 (5.3%) | 15 (11.4%) | 4 (3.0%) | 0.674 |
| Blood transfusion supplies | 56 (42.4%) | 12 (9.1%) | 36 (27.3%) | 27 (20.5%) | 1 (0.8%) | 0.534 |
| **Post-abortion care supplies** |  |  |  |  |  |  |
| Misoprostol | 31 (23.5%) | 12 (9.1%) | 52 (39.4%) | 36 (27.3%) | 1 (0.8%) | 0.305 |
| Manual vacuum aspirator | 6 (4.5%) | 2 (1.5%) | 24 (18.2%) | 100 (75.8%) | 0 (0.0%) | 0.072 |
| Parenteral antibiotics | 0 (0.0%) | 1 (0.8%) | 30 (22.7%) | 101 (76.5%) | 0 (0.0%) | 0.024 |
| Local anesthetic | 1 (0.8%) | 3 (2.3%) | 10 (7.6%) | 118 (89.4%) | 0 (0.0%) | 0.000 |
| Speculum and tenaculum | 1 (0.8%) | 1 (0.8%) | 3 (2.3%) | 126 (95.5%) | 1 (0.8%) | 0.000 |
| Sharp curettage tray | 49 (37.1%) | 7 (5.3%) | 15 (11.4%) | 49 (37.1%) | 12 (9.1%) | 0.148 |
| **Neonatal resuscitation supplies** |  |  |  |  |  |  |
| Thermometer | 0 (0.0%) | 1 (0.8%) | 18 (13.6%) | 113 (85.6%) | 0 (0.0%) | 0.092 |
| Pulse oximeter | 47 (35.6%) | 13 (9.8%) | 19 (14.4%) | 53 (40.2%) | 0 (0.0%) | 0.353 |
| Bulb syringe | 5 (3.8%) | 0 (0.0%) | 10 (7.6%) | 115 (87.1%) | 2 (1.5%) | 0.099 |
| Suction catheter | 2 (1.5%) | 2 (1.5%) | 20 (15.2%) | 108 (81.8%) | 0 (0.0%) | 0.008 |
| Infant size bag and mask | 2 (1.5%) | 2 (1.5%) | 3 (2.3%) | 125 (94.7%) | 0 (0.0%) | 0.000 |
| Nasogastric tube | 2 (1.5%) | 1 (0.8%) | 29 (22.0%) | 99 (75.0%) | 1 (0.8%) | 0.050 |
| Laryngoscope and endotracheal tube | 64 (48.5%) | 11 (8.3%) | 26 (19.7%) | 29 (22.0%) | 2 (1.5%) | 0.006 |
| Resuscitation table with heat source | 17 (12.9%) | 0 (0.0%) | 3 (2.3%) | 112 (84.8%) | 0 (0.0%) | 0.808 |
