# Supplementary material for: Clinical readiness for essential maternal and child health services in Kenya: A cross-sectional survey
Source: PLOS Glob Public Health. 2023 Dec 15;3(12):e0002695. doi: 10.1371/journal.pgph.0002695 (PMC10723700; doi:10.1371/journal.pgph.0002695)
Supplement: S3 Table — (DOCX) [file pgph.0002695.s003.docx]

**S3 Table.** **Provider and Facility Level Factor Associations with Individual MCH Topics by Generalized Estimating Equations.**

| **Provider and Facility Level Factors** | **Interaction Between Factor and MCH Topics (p-value)** |
| --- | --- |
| **Facility Location** | 0.061 |
| **Facility Type** | 0.226 |
| **Provider Age** | 0.764 |
| **Provider Sex** | 0.859 |
| **Provider Role** | 0.054 |
| **Provider Years in Role** | 0.771 |
| **Provider Years Since Initial Training** | 0.911 |
| **Number of Trainings** | 0.787 |
| **Number of Recent Trainings** | 0.934 |
| **Supply Availability (aggregate)** | 0.911 |
