# Supplementary material for: Clinical readiness for essential maternal and child health services in Kenya: A cross-sectional survey
Source: PLOS Glob Public Health. 2023 Dec 15;3(12):e0002695. doi: 10.1371/journal.pgph.0002695 (PMC10723700; doi:10.1371/journal.pgph.0002695)
Supplement: Attachment — Submitted filename: Kenya MCH_PLoS_Reviewer Comments_FINAL.docx [file pgph.0002695.s004.docx]

**Clinical Knowledge Among Health Care Providers of Essential Maternal and Child Health Services in Kenya: A Cross-Sectional Survey**

***Editor Comments:***

1. Please ensure that Funding Information and Financial Disclosure Statement are matched.

Thank you. The Funding Information has been added to the title page (lines 18-25) to match the Financial Disclosure Statement.

***Reviewers' Comments:***

Reviewer's Responses to Questions

*Comments to the Author*

*1. Does this manuscript meet PLOS Global Public Health’s publication criteria? Is the manuscript technically sound, and do the data support the conclusions? The manuscript must describe methodologically and ethically rigorous research with conclusions that are appropriately drawn based on the data presented.*

*Reviewer #1: Yes*

*Reviewer #2: Partly*

*2. Has the statistical analysis been performed appropriately and rigorously?*

*Reviewer #1: Yes*

*Reviewer #2: Yes*

*3. Have the authors made all data underlying the findings in their manuscript fully available (please refer to the Data Availability Statement at the start of the manuscript PDF file)?*

*The PLOS Data policy requires authors to make all data underlying the findings described in their manuscript fully available without restriction, with rare exception. The data should be provided as part of the manuscript or its supporting information, or deposited to a public repository. For example, in addition to summary statistics, the data points behind means, medians and variance measures should be available. If there are restrictions on publicly sharing data—e.g. participant privacy or use of data from a third party—those must be specified.*

*Reviewer #1: Yes*

*Reviewer #2: Yes*

*4. Is the manuscript presented in an intelligible fashion and written in standard English?*

*PLOS Global Public Health does not copyedit accepted manuscripts, so the language in submitted articles must be clear, correct, and unambiguous. Any typographical or grammatical errors should be corrected at revision, so please note any specific errors here.*

*Reviewer #1: Yes*

*Reviewer #2: Yes*

*5. Review Comments to the Author*

*Please use the space provided to explain your answers to the questions above. You may also include additional comments for the author, including concerns about dual publication, research ethics, or publication ethics. (Please upload your review as an attachment if it exceeds 20,000 characters)*

Reviewer #1:

*Dear Author*

*Thanks for your submission*

*It is an interesting paper but needs some work on clarity*

*I think you need to make it very clear throughout the abstract, introduction and methods that you are looking at overall knowledge re medical emergencies and then the knowledge of each of them*

*While you trialled a questionnaire about post abortion care as you did not test validity, reliability etc of this I would just state that you needed to design your own methods to collect this data ( and address limitations of this in limitation section of discussion) and then focus on describing knowledge etc across all and each medical emergencies*

*Otherwise the reader is confused as to whether this is a paper on postabortion care in which case a different introduction is required or medical emergencies for MCH ( I would go with the latter)*

Thank you for your suggestions regarding the manuscript. We agree that trialing the post-abortion care questionnaire is a more accurate description as we did not have the number of subjects or questions to do a thorough test of the validity and reliability of this questionnaire. We have changed the wording on line 107: “Our secondary aim was to trial a case-based questionnaire on post-abortion care given the gaps in the literature.” We also expanded on our analysis to indicate this as well in lines 214-215: “As this is the first trial of these post-abortion care case vignettes, these analyses were exploratory only. Rigorous validation of this instrument was beyond the scope of this study.”

*In the Intro line 77 could you please give some information on what were the results from 12 and 13. I would also suggest considering a strengths based where possible approach to reporting results and where are clinicians doing well ?*

Thank you for your comment. We have added additional information regarding the results from citations 12 and 13 on lines 91-94: “When case-based questionnaires were used to assess knowledge acquisition following team training and simulation learning on a variety of MCH emergencies, improvements in clinical knowledge were seen from pre-training to post-training [12,13].”

We have also added information to the discussion highlighting the fact that simulation-based training does improve clinical knowledge and has been shown across a number of LMIC in lines 351-353: “Prior studies have indicated that trainings, and specifically simulation-based training, in MCH topics has resulted in improved knowledge on treating MCH emergencies in a variety of LMIC settings [26].”

*In the methods please use headings and separate out outcome measures from analysis as it is currently mixed together and confusing*

Thank you for your request. We have split the previously named “Statistical Methods” into “Outcome Measures” and “Statistical Analysis” headers to differentiate the different parts of this section.

*Please give a reference and short sentences on the validation of PRONTO training questions*

Thank you for your request. We have updated the information on the PRONTO training questionnaires in the Data Collection section of the manuscript. Lines 145-147 now read: “No formal validation process was followed, but these knowledge questionnaires have been tested across multiple low- and middle-income countries worldwide [12, 13, 23, 24].”

*Results*

*I am confused why 95% confidence intervals are not used when reporting the outcomes in Table 3?*

Table 3 is a purely descriptive table that reports the mean and standard deviation for participant perceived supply availability across the MCH topics among our study population. As our sample was a convenience sample and not necessarily reflective of all individuals providing MCH care in western Kenya, we did not calculate 95% confidence intervals for a broader population.

*I would like to see clearer recommendations for services and research as a result of your findings that are feasible in a LMIC setting*

Thank you for your comment. We have added an additional lines (401-403) in the conclusions to make our recommendations more clear: “Attention to rigorous training programs, especially simulation-based training, can help improve provider knowledge; and care should be made to focus on improving access to supplies needed for basic EmOC interventions.”

*Reviewer #2: The manuscript is an interesting read, however the title is not quite clear. The body of the manuscript carries a lot of observations that are not related to the title of study. It requires modification of the title to make it as comprehensive as much as possible comprehensive.*

Thank you for your thoughts regarding the title of the manuscript. The title of the manuscript has been changed to “Clinical readiness for essential maternal and child health services in Kenya: A cross-sectional survey” to encompass the additional observations not directly related to clinician knowledge such as training and supply availability.

*6. PLOS authors have the option to publish the peer review history of their article (what does this mean?). If published, this will include your full peer review and any attached files.*

*Do you want your identity to be public for this peer review? If you choose “no”, your identity will remain anonymous but your review may still be made public.*

*For information about this choice, including consent withdrawal, please see our Privacy Policy.*

*Reviewer #1: No*

*Reviewer #2: Yes: Dr. Angeline C. Kirui, RN, PhD*

*Reviewer comments for the manuscript” PGPH-D-22-01510*
**Clinical knowledge among health care providers of essential maternal and child health services in Kenya: A cross-sectional survey**; *PLOS Global Public Health”*.

***Summary of reviewer comments***

***Title & introduction***: The topical area of study is of great significance in public health. The justification is well stated however the title is not comprehensive.

***Methods***: The study design is appropriate, but the sample size is very small hence limited generalization of findings. The non probability convenience sampling method adopted reduced the representativeness of the study sample.

**Statistical *analyses***: Both descriptive and some inferential statistics were presented.

***Conclusions***: conclusions are based on data provided

***Tables:*** Well labelled and adequate to support the study findings

***Detailed reviewer comments***

1. *Line-1. The title lacks clarity*

*The title is not clear whether it is clinical knowledge on care or plus understanding on*

*the essential supplies for different types of maternal and neonatal care.*

Thank you for your comment. As indicated above, we have changed the title to “Clinical readiness for essential maternal and neonatal care services in Kenya: A cross-sectional survey” to indicate the broad areas of clinician knowledge, training and supply availability detailed in the manuscript.

2. *Line-26. The additional sub objective of validation of post abortion care questionnaire is misplaced and is not captured in the title of the manuscript.*

Thank you for your comment. We piloted our post-abortion care case-based questions; however, validation of this tool was beyond the scope of this project given the small sample size. As multiple maternal and child health emergencies were investigated, we chose to keep the title broad to encompass all of the information presented, not just that related to post-abortion care.

3. *Line 33. The sample size is too small for the 37 facilities. Some cadres of service providers were not adequately represented hence risk of bias in study findings. Nurses were the explicit majority as compared to clinical and medical officers.*

Thank you for your comment regarding sample size. Most of the facilities where this survey were administered were level 4 health facilities (sub-County Hospitals), which are primarily staffed by nurses and nursing officers. Only level 5 health facilities (County Hospitals) have regular clinical and medical officer staff; which made up 3 of the 37 facilities. One hundred and thirty-two participants were interviewed, or an average of 3.5 participants per site. Often only two to four service providers were working at any time in Maternal and Child Health, which is why the average number of participants per site was 3.5. Convenience sampling among providers is noted as a limitation in the discussion.

4. *Line 36. The observation that “All facilities had access to general supplies” is ectopic to the study in reference to the title of the manuscript, unless the title is amended accordingly.*

Thank you for your comment. As noted above, we have changed the title to reflect the manuscript’s overall goal of reporting clinical readiness, not just provider knowledge. We report on both general supply availability as well as supplies necessary for the treatment of the specific maternal and child health emergencies detailed in the paper (post-abortion care, pre-eclampsia, postpartum hemorrhage, neonatal resuscitation).

5. *Line 40. The observation that “there were gaps in supply availability” not related to title of study unless amended to include the same.*

Thank you. As noted above, we have changed the title of the study to be more broadly applicable.

6. *Line-41” The author has not demonstrated relationship between training and reported clinical knowledge in participants” There is need for comparative analysis between the two variables*

Thank you for your comment. In the results section on clinical knowledge, we do note that: “Training in an increasing number of MCH topic areas (regardless of specific training) was associated with increased clinical knowledge. Training in one, two, three, or four MCH topics was associated with a 6.0% (95% CI = -0.09, 12.2), 8.3% (95% CI = 1.9, 14.8), 8.4% (95% CI = 2.5, 14.3), and 11.2% (95% CI = 5.2, 17.2) increase in the percentage of correct questions across all topics tested (p = 0.03), respectively.” (lines 283-287). We also had a model comparing clinical knowledge scores was adjusted for level of training (number of MCH topics ever trained on, number of MCH topics trained on within the last 3 years, and years since completing medical training). This adjusted model had the same estimates and p-value as the unadjusted model reported in Table 5. We have clarified this further in lines 296-300: “Estimates and p-values were the same for the unadjusted model and all three adjusted models: one adjusting for demographics (facility county and level, provider role, age, sex, years in role); one adjusting for training (number of MCH topics ever trained on, number of MCH topics trained on within the last 3 years, years since completing medical training); and one adjusting for supply availability.”

7. *Line-46. The statement of ‘rising age in motherhood’ does not reflect the true picture in the region where this study was carried out. The truth is there is a high prevalence of teenage pregnancies marked with high risk of abortion and poor maternal and neonatal outcomes.*

Thank you for your comment. We agree that teen pregnancy is also a challenge in western Kenya, with the latest Kenya Demographic and Health Survey from 2022 reporting that 15% of individuals aged 15-19 had ever been pregnant. However, the population in Kenya has been aging overall with an increased proportion of individuals with non-communicable diseases, which may affect maternal and neonatal outcomes. We have revised our introduction to talk more generally about the extremes of ages in both directions – both older pregnancies and teen pregnancies – as both carry increased risks associated with poor maternal and neonatal outcomes. Lines 54-60 now read: “Population aging and increasing rates of non-communicable diseases (NCDs) among pregnant women have elevated the risk of morbidity and mortality during pregnancy and childbirth in low- and middle-income countries (LMIC). At the same time, LMIC continue to have high rates of teen pregnancies. LMIC also struggle to respond to obstetric-related emergencies, which are more likely in high-risk pregnancies. Extremes in age of motherhood (both younger and older) are associated with hypertensive diseases, gestational diabetes, fetal macrosomia, miscarriage, and stillbirth [1].”

8. *Line -80. The author gives more focus on post abortion care topic and refers to it more in the entire manuscript as compared to the other topics of Pre-eclampsia, Postpartum hemorrhage and Neonatal resuscitation. The overemphasis introduces bias in dissemination of study findings*

Thank you for your comment. As less is known about post-abortion care in this setting compared to the other MCH topics, particularly in the setting of the second aim to pilot a case-based questionnaire on post abortion care, we focused more on this outcome in our manuscript. However, we believe that we still discussed the other topics in depth to provide a full picture of clinical readiness on all topics.

9. *Line 92. Author talks of “pilot of a case-based questionnaire on post abortion care”. The collection of data on post abortion care using a non-validated questionnaire may have introduced bias as compared to the other topical areas that had already validated generic questionnaires adopted for use in data collection*

Thank you for your comment regarding the pilot case-based questionnaire on post-abortion care. As you mention, this is a source of bias in our results, as the other three sections of the clinical knowledge survey were previously validated. We do indicate this bias in our conclusions (lines 392-394: “Some of the differences in post-abortion care knowledge may be due to potential different difficulty level of these questions compared to the other clinical knowledge questions.”). We did complete a factor analysis to determine if specific questions were most useful in assessing knowledge on post-abortion care, but no specific questions showed statistical significance.

10. *Line 115.” Participants completed verbally administered interviews” by who? where? How was data captured? Data collection process isn’t clear.*

Thank you for your question. We have clarified the data collection process in lines 137-141: “Providers were read survey questions by members of the study team in a private location at each of the study health facilities. Answers were recorded on either paper surveys or handheld tablets and later uploaded to a centralized secure Qualtrics database (SAP, Walldorf, Germany), which was then transferred to a secure server to use for data analysis.”

11. *Line 154.” assess differences in supply availability between facilities”. Which objective is being described by the author? There is need to revise the title and sub objectives to include the topic on supply availability.*

Thank you. As noted above, we have changed the title of the study to be more broadly applicable.

12. *Line 166-168. Author describes the pilot of post abortion care questionnaire.*

*Reviewer is of the view that Validation of post abortion care questionnaires needed an independent study. The validation process presents a study within a study phenomenon”.*

Thank you for your comment. We agree that a thorough validation process of the post abortion care questions necessitates an independent study. As this was the first time this questionnaire had been used, we completed a factor analysis and latent class analysis to see if we could identify any questions for further study in a broader context. We have revised the methodology to confirm that this element of the study was exploratory only and further study is needed to validate the results. We note in lines 214-215 “As this is the first trial of these post-abortion care case vignettes, these analyses were exploratory only. Rigorous validation of this instrument was beyond the scope of this study.”

13. *Line 180. Table 1. Typo error- ‘provide role’ should read ‘provider role’*

Thank you for the correction. The line has been changed to “Provider Role”.

14. *Line 215-220. The author compared the availability of supplies by level of health facility i.e., Sub- County versus county and failed to analyze the average service providers’ clinical knowledge by level of facility. The assessment on availability of supplies was not part of the objectives of the study at inception.*

Thank you for your comment. We did report the differences by health facility level briefly in the section on clinical knowledge: “Mean composite scores were not statistically different by facility level or provider role (mean percentage correct 63.7% among medical and clinical officers versus 63.2% among nurses, p = 0.87; mean percentage correct 63.4% in sub-county hospitals versus 62.8% in county hospitals, p = 0.87).” (lines 280-283), indicating no statistically significant difference by sub-county versus county health facility. A model comparing clinical knowledge scores was adjusted for demographics, including facility county and level of facility. This adjusted model had the same estimates and p-value as the unadjusted model reported in Table 5. As mentioned previously, we have clarified this further in lines 296-300: “Estimates and p-values were the same for the unadjusted model and all three adjusted models: one adjusting for demographics (facility county and level, provider role, age, sex, years in role); one adjusting for training (number of MCH topics ever trained on, number of MCH topics trained on within the last 3 years, years since completing medical training); and one adjusting for supply availability.”

We have also updated the aim of the study in the introduction to indicate the more broad objectives of this paper. Lines 103-107 now read: “The aim of this study was to determine clinical readiness among health care providers in three counties in western Kenya of four MCH topics: post-abortion care, management of pre-eclampsia and eclampsia, management of postpartum hemorrhage and neonatal resuscitation through understanding clinical knowledge, training and supply availability for each MCH topic.”

***Note****: I propose an appropriate title for the manuscript to read” Clinical readiness for essential maternal and neonatal care services in Kenya. A cross sectional survey.*

***Sub objectives****- Providers’ clinical knowledge, training & Supplies availability.*

Thank you. We have changed the title as indicated above.
