# Supplementary material for: Clinical readiness for essential maternal and child health services in Kenya: A cross-sectional survey
Source: PLOS Glob Public Health. 2023 Dec 15;3(12):e0002695. doi: 10.1371/journal.pgph.0002695 (PMC10723700; doi:10.1371/journal.pgph.0002695)
Supplement: Attachment — Submitted filename: Kenya MCH_PLoS_Reviewer Comments_V2_FINAL.docx [file pgph.0002695.s005.docx]

**Clinical Knowledge Among Health Care Providers of Essential Maternal and Child Health Services in Kenya: A Cross-Sectional Survey**

***Reviewers' Comments:***

Reviewer's Responses to Questions

*1. If the authors have adequately addressed your comments raised in a previous round of review and you feel that this manuscript is now acceptable for publication, you may indicate that here to bypass the “Comments to the Author” section, enter your conflict of interest statement in the “Confidential to Editor” section, and submit your "Accept" recommendation.*

*Reviewer #2: All comments have been addressed*

*Reviewer #3: (No Response)*

*2. Does this manuscript meet PLOS Global Public Health’s publication criteria? Is the manuscript technically sound, and do the data support the conclusions? The manuscript must describe methodologically and ethically rigorous research with conclusions that are appropriately drawn based on the data presented.*

*Reviewer #2: Yes*

*Reviewer #3: Partly*

*3. Has the statistical analysis been performed appropriately and rigorously?*

*Reviewer #2: Yes*

*Reviewer #3: No*

*4. Have the authors made all data underlying the findings in their manuscript fully available (please refer to the Data Availability Statement at the start of the manuscript PDF file)?*

*The PLOS Data policy requires authors to make all data underlying the findings described in their manuscript fully available without restriction, with rare exception. The data should be provided as part of the manuscript or its supporting information, or deposited to a public repository. For example, in addition to summary statistics, the data points behind means, medians and variance measures should be available. If there are restrictions on publicly sharing data—e.g. participant privacy or use of data from a third party—those must be specified.*

*Reviewer #2: Yes*

*Reviewer #3: No*

As detailed in the submission under Data Availability, the data that support the findings of this study are openly available in the Duke Digital Repository for Research Data at http://doi.org/10.7924/r47m0fd7k.

*5. Is the manuscript presented in an intelligible fashion and written in standard English?*

*PLOS Global Public Health does not copyedit accepted manuscripts, so the language in submitted articles must be clear, correct, and unambiguous. Any typographical or grammatical errors should be corrected at revision, so please note any specific errors here.*

*Reviewer #2: Yes*

*Reviewer #3: Yes*

*6. Review Comments to the Author*

*Please use the space provided to explain your answers to the questions above. You may also include additional comments for the author, including concerns about dual publication, research ethics, or publication ethics. (Please upload your review as an attachment if it exceeds 20,000 characters)*

*Reviewer #2: Clinical readiness for essential maternal and child health services in Kenya: A cross-sectional survey*

*Thank you for the opportunity to review this paper*

*• The manuscript covers a significant health topic*

*• The quality of manuscript is good*

*• It meets the minimum requirements for scientific writing*

*Recommendation*

*• The manuscript to be considered for publication after authors’ attention to the review comments highlighted to the satisfaction of journal editor*

*Specific comments for authors’ consideration*

*Line 47… substitute the word “supply” with the word …” supplies” ……*

Thank you for your suggestion. As the word “supply” is in the context of “supply availability,” we believe it should stay as previously worded.

*Line 48, reorganize the sentence to read…” decrease pregnancy and postpartum complications”.*

Thank you for your comment. We have reorganized the sentence to read: “Increased training on MCH topics may decrease pregnancy and postpartum complications.”

*In the introduction line 53, authors should give teen pregnancies more weight as an independent variable besides population aging and increase in NCDs as a determinant of need for emergency obstetric care in LMIC. Authors to review the first sentence to read; Teen pregnancies, aging population and increase in non-communicable diseases………. delete the sentence on teen pregnancy in line 55 & 56*

Thank you for your suggestion to place more weight on teen pregnancies. We have re-written the introduction to state: “Pregnancy at the extremes of age (both increased teen pregnancy and pregnancy at advanced maternal age) and increasing rates of non-communicable diseases (NCDs) among pregnant women have elevated the risk of morbidity and mortality during pregnancy and childbirth in low- and middle-income countries (LMIC).” We have also deleted the sentence on teen pregnancy in line 55-56 as you had indicated.

*Line 70. Replace the word “these goals” with “the goals” …...*

We appreciate the reviewer’s suggestion. The sentence now reads: “The goal to provide essential MCH services aligns with broader recommendations to ensure that emergency obstetric care is available to all women and newborns.”

*In Line 74, specify the providers to read “health care providers”*

Thank you for your comment. We have specified that providers are “health care providers” in this sentence.

*Line 124 & 125 …participants were excluded if they could not fill the survey questionnaire...; while line 126… reads of verbally administered interviews by study person; Authors to clarify the actual data collection tool adopted and procedure of data collection*

We appreciate the reviewer’s comments. We have revised the sentence for clarity: “Participants were excluded if they could not provide informed consent or respond to the survey questionnaire in English.” We utilized verbally administered questionnaires as detailed in the subsequent lines.

*Line 151…. and line 153… replace ….” supply” with “supplies”*

Thank you for your suggestions. As above, because the word “supply” is in the context of “supply availability,” we believe it should stay as written.

*Line 165…. The word “Supply” should be replaced with the… “type of supplies or category of supplies”. The author should replace the action verb supply with supplies throughout the document. Line 216, Table 3 column 1, row 1; line 220, 226, 231, 247, 265, 342, 347…. Etc*

We appreciate your comment. We have re-written the sentence to state: “Variability in the availability of each type or category of supplies was divided into within facility and between facility variability and reported as an intraclass correlation coefficient (ICC) to assess differences in supply availability between facilities. All other notations of supplies are in the context of supply availability and should be kept as they are.

*Line 250. Qualify…...’providers’ as “health care providers”*

Thank you for the comment. We have revised the sentence to indicate that we are referring to “health care providers.”

*Line 251. Add the article ‘the’ before providers to read... “the providers”*

We appreciate the comment. We have added the article “the” to note “The providers tended to score highest on the post-abortion care questions with a mean (SD) score of 71.6% (16.7%), followed by postpartum hemorrhage at 69.6% (21.2%), neonatal resuscitation at 56.8% (19.1%), and lastly pre-eclampsia and eclampsia at 55.3% (20.5%) (Table 5).”

*Line 275 to 277 carries a biased observation that adversely depicts the nurses as poor performers in post abortion when indeed the sampling was skewed and finding is not statistically significant. Authors to review the statement…..*

Thank you for your suggestion. Given that this information was not statistically significant and to not introduce any biased reporting, we have revised the sentence to read: “The worst performing group size was too small to determine any facility or provider characteristics associated with poor performance (Table 6).”

*.*

*Line 286… specify providers to read … “health care providers” ….*

We appreciate the suggestion. We have added “health care providers” for clarity.

*Line 286… substitute the word …” decreasing” ... with... “reducing”*

Thank you for the comment. We have changed the sentence to read: “Facility and health care provider readiness to respond to MCH emergencies are key in reducing maternal mortality.”

*Line 315, add the word ‘only’…..the statement should read partly .. legalized abortion only……..to specify when the law allows procurement of abortion*

We appreciate the comment. We have revised the sentence to add the word “only” for clarity: “This may be due to a temporary halt in documentation of standards and guidelines on how to provide appropriate post-abortion care services following changes to the Kenyan Constitution in 2010, which legalized abortion only when the life or health of a woman are in danger [26].”

*Line 317… replace the word …” decreasing” … with “…...reducing”*

Thank you for your suggestion. We have rewritten this sentence with this change: “Many providers did not know how to interpret these legal changes in their scope of practice, reducing the amount of formal training in this area [27].”

*Line 356... review the statement that reads ...” supply availability” to read ….” supplies availability” or “availability of supplies”*

We appreciate your comment. As noted earlier, we have been using “supply availability” for consistency throughout the paper and believe this is the correct utilization of this term.

*References No. 30 & 31 have not been cited in the text… Authors to drop them or cite where applicable*

Thank you for your comment. We have revised the reference numbers to match the citations as appropriate.

*Reviewer #3: Dear author,*

*The paper is insightful and introduces a new area of focus, however it needs some work on clarity as well as further improvements.*

*1. It is not clear what the objective of the paper is. It is not clear if the paper seeks to determine clinical readiness or just simply seeks to understand MCH care provision in the three counties. This has not been presented in a clear manner. From the title the focus is to determine clinical readiness among health care providers (line 99) but how this is done or has been done does not clearly come out in the paper. In line 102, the approach is stated but there is a lack of how clinical knowledge, training, and supply availability determine clinical readiness or how they link up.*

Thank you for your suggestion regarding clarity for this paper. We have added additional information to the introduction as follows:

“To ensure that women receive the quality care during pregnancy and delivery that will improve maternal morbidity and mortality, it is necessary to understand not only facility availability of supplies and services but also training and clinical knowledge of healthcare professionals. While prior studies have increased the specificity of measuring clinical readiness through identifying resources to identify, treat, and monitor or modify MCH emergencies at different health facilities; they lack information on whether providers have been trained to use these supplies or have current knowledge on how to use these supplies [9,10]. The aim of this study was to further improve the accuracy in the diagnosis of clinical readiness by measuring not just supply availability, but also an assessment of provider training and knowledge. We sought to do this across four MCH topics: post-abortion care, management of pre-eclampsia and eclampsia, management of postpartum hemorrhage and neonatal resuscitation. Our secondary aim was to trial a case-based questionnaire on post-abortion care given the gaps in the literature.”

*2. Similarly, from my understanding, there is no outcome measure for clinical readiness (lines 150 -159), which begs the question what are the independent and dependent variables in this analysis and is there a framework that guides the study ? If so, it will be better to include it in the narrative in order to give direction or tie up all parts of the study in a more coherent manner.*

We appreciate your comment. As mentioned in the revised introduction, we did not seek to put together a composite score for clinical readiness given the heterogeneity of data across all the sites. Instead, we sought to enhance the current standard of defining clinical readiness through supply availability by adding provider training and knowledge across the four MCH topics. We used the Demographic Health Survey Service Provision Assessment and a clinical cascade model for supply availability (references 9-10) to determine the supply availability questions; and used a knowledge, attitudes and practices (KAP) framework for the provider knowledge and training questions.

*3. In line 136-137 there is a contradiction, on one hand there is the claim that the questionnaires were validated by experts and then in the line that follows you indicate that no formal validation process was followed. This does not make sense, either validation was done or not but not both. By design, if a questionnaire has been applied and tested multiple times then there is some form of intrinsic validity in their use whether all or some of the questions from the questionnaire are used in the study.*

Thank you for your comment and desire for clarity on this point. We have revised this section to read: “Knowledge questionnaires from PRONTO were developed by the study team and reviewed by experts prior to their use in assessing clinical knowledge among health care providers. No formal validation process was followed, but these knowledge questionnaires have been tested across multiple low- and middle-income countries worldwide [12, 13, 23, 24].” As noted from the sentence above, there was no specific validation process for the PRONTO clinical knowledge questions, but as you mentioned, there is some intrinsic validity in their use given that they have been applied and tested many times across multiple clinical contexts in low- and middle-income countries.

*4. Even though assessing clinical knowledge, training and resources independently provides valuable insights the study would be better if all this can be linked up to clinical readiness at the facility or individual level. As indicated in the paper the study was exploratory in nature and thus a simple linear model or a likelihood model would have sufficed in linking clinical readiness to the exploratory variables . The sample size (n=132) is sufficient to ensure convergence even if the study was to rely on a maximum likelihood estimation approach of overall clinical readiness either at the facility level or individual level. Also, the importance of comparing the correct questions between MCH topics with post abortion care chosen as the reference has not been clearly elucidated and its not clear why this was done.*

We appreciate the reviewer’s suggestions. Our study was exploratory in nature, and thus the information we provided regarding supply availability, training and clinical knowledge were presented individually in a descriptive manner. We did adjust our models investigating differences in clinical knowledge by MCH topic by both provider training and supply availability, neither of which changed our overall estimates of clinical knowledge (Table 5). Further, we investigated the effect of both provider and facility level factors on clinical knowledge specifically looking at questions around diagnosis, treatment, or monitoring of the MCH emergency as indicated in lines 318-326: “Finally, clinical knowledge questions across all four MCH topics were separated by question type (asking about diagnosis, treatment, or monitoring obstetric or neonatal emergency) and correlated with provider and facility factors. Male providers scored on average 8.9% (66.1% versus 57.1%; 95% CI = 2.3, 15.6; p = 0.009) higher on questions regarding how to monitor emergencies. Age was also significantly associated with knowledge on how to monitor emergencies (p = 0.037). The relationship was non-linear so linear splines were created with a knot at 35 years. There was not a statistically significant association when age was less than or equal to 35 years. However, beyond 35 years, a five-year increase was associated with a 4.2% reduction in the percentage of correct questions.”

In order to have a true measure of clinical readiness, we would want to correlate our assessment of clinical knowledge, training and resources on specific clinical outcomes (e.g. morbidity and mortality associated with postpartum hemorrhage, pre-eclampsia/eclampsia, incomplete abortion or low APGAR scores at birth), which was beyond the scope of our project. Additionally, we had more inter-provider variability in our responses than anticipated, which made aggregate measures at a facility level extremely challenging.

In terms of our models for clinical knowledge on the different MCH topics, we fit a linear mixed model with the proportion of correct questions as the outcome and MCH topic as an independent variable. Responses, one score for each MCH topic, were clustered within providers and providers were clustered within facilities. This was achieved by including a random effect for participant and facility.

Given MCH topic was an independent variable in this model, we had to choose a reference category. Post-abortion care was chosen as the reference because those questions were generated by our study team and not previously utilized by the PRONTO team to determine if there was any difference between our questions and those that already had intrinsic validity.

The goals of this particular analysis was to (1) compare the proportion of correct questions between topics to better understand if providers are, on average, better trained in one topic than another and (2) if certain individual or facility characteristics influenced knowledge. The result was that providers tended to score significantly better on the post-abortion care questions than the neonatal resuscitation and pre-eclampsia/eclampsia questions. We highlight reasons why this may be the case in lines 380-387 in the discussion. We also found that the number of MCH topics trained in was correlated with overall increased knowledge, as noted in lines 267-271.

*5. Linear models with fixed effects require evidence of non-independence, heterogeneity and non-linearity within the data which has not been presented or alluded to within the paper. It also assumes that the explanatory variables are linearly related to the response. If there is evidence for any of this, then this should be included and clarified presented as a justification.*

Thank you for your comments. A linear mixed model was chosen to compare the proportion of correct questions between MCH topics because responses, one score for each MCH topic, were clustered within participants and participants were clustered with facility. Responses given by the same participant are correlated and responses from providers within the same facility are also likely to be correlated. The linearity assumption for continuous covariates was assessed using a lack-of-fit test comparing the linear fit to a restricted cubic spline fit with knots placed at the 10^th^, 50^th^, and 90^th^ percentiles. If a significant non-linear relationship was observed, then a plot with the restricted cubic spline fit was examined to determine an appropriate knot for linear splines. This has been clarified in the methods, lines 197-201.

*6. The statistical methodology applied is disjointed and fails to clearly address the objectives of the paper and thus the paper is not replicable in the same context elsewhere. It also makes it difficult to determine if the conclusions have been arrived at in an objective manner from the findings. From the paper its alluded that clinical readiness outcome might not be a continuous variable but dichotomous in nature. Considerations should therefore be made to develop a statistical model that links clinical readiness to training, resources, and clinical knowledge as that would make for a better paper.*

We appreciate these comments from the reviewer. Please see the above answers to questions 4 and 5 regarding the statistical methodology for this paper. As previously mentioned, this paper was exploratory in nature, and given the heterogeneity of responses, we were not able to create a single score indicating clinical readiness. For example, different providers at the same facility might note that supplies were always available versus less available or might note that many providers were trained in a specific MCH topic versus few providers. We know from prior literature that signal functions (presence or absence of a specific supply – e.g. oxytocin for postpartum hemorrhage) are less accurate at predicting clinical readiness, and thus the focus on clinical cascades to identify supplies that identify the MCH emergency, treat the condition and then monitor the condition are more accurate [9,10]. We believe that additional information on not only supply availability but if health care personnel were trained to use the supply as well as clinical knowledge to use the supply also tie into clinical readiness. Further study to link our findings to specific clinical outcomes could help identify what might be included in the statistical model.

*7. PLOS authors have the option to publish the peer review history of their article (what does this mean?). If published, this will include your full peer review and any attached files.*

Yes, we accept publishing the peer review history of the article.

*Do you want your identity to be public for this peer review? If you choose “no”, your identity will remain anonymous but your review may still be made public.*

*For information about this choice, including consent withdrawal, please see our Privacy Policy.*

*Reviewer #2: Yes: DR. Angeline Chepchirchir Kirui*

*Reviewer #3: No*
