# Supplementary material for: Clinical readiness for essential maternal and child health services in Kenya: A cross-sectional survey
Source: PLOS Glob Public Health. 2023 Dec 15;3(12):e0002695. doi: 10.1371/journal.pgph.0002695 (PMC10723700; doi:10.1371/journal.pgph.0002695)
Supplement: Attachment — Submitted filename: 2nd Review comments Plos Clinical readines for MCH.docx [file pgph.0002695.s006.docx]

**2^nd^ Review comments**

**Manuscript title**: Clinical readiness for essential maternal and child health services in Kenya: A cross-sectional survey

- The manuscript has been revised based on the proposed suggestions and comments on 1^st^ review.
- The authors agreed to most of the suggested review comments but declined one or two i.e supply versus supplies and made clarification on the intended meaning while retaining the original words
- The clarity of the manuscript has improved greatly

**Recommendation**

- Accept the manuscript for publication

Thank you

Dr. Angeline Chepchirchir Kirui

Reviewer.
